# An intuitionistic fuzzy entropy approach for supplier selection

**DOI:** 10.1007/s40747-020-00224-6

**Published:** 2021-05-05

**Authors:** Mohamadtaghi Rahimi, Pranesh Kumar, Behzad Moomivand, Gholamhosein Yari

**Affiliations:** 1 Department of Mathematics and Statistics, University of Northern British Columbia, Prince George, BC Canada; 2 Department of Management, Qom Branch, Islamic Azad University, Qom, Iran; 3 Department of Mathematics, Iran University of Science and Technology, Tehran, Iran

**Keywords:** Multicriteria decision-making, Supplier selection, Intuitionistic fuzzy entropy, Intuitionistic fuzzy set

## Abstract

Due to apparent flexibility of Intuitionistic Fuzzy Set (IFS) concepts in dealing with the imprecision or uncertainty, these are proving to be quite useful in many application areas for a more human consistent reasoning under imperfectly defined facts and imprecise knowledge. In this paper, we apply notions of entropy and intuitionistic fuzzy sets to present a new fuzzy decision-making approach called intuitionistic fuzzy entropy measure for selection and ranking the suppliers with respect to the attributes. An entropy-based model is formulated and applied to a real case study aiming to examine the rankings of suppliers. Furthermore, the weights for each alternative, with respect to the criteria, are calculated using intuitionistic fuzzy entropy measure. The supplier with the highest weight is selected as the best alternative. This proposed model helps the decision-makers in better understanding of the weight of each criterion without relying on the mere expertise.

## Introduction

Decision-maker’s judgments, including preference information, are usually stated in linguistic terms. There are many approaches proposed for modeling the decision linguistic term sets. Zadeh [43–45] defined the linguistic variable as a variable whose values are words or sentences in a natural or artificial language in his three consecutive papers. Recently, Morente-Molinera et al. [23] provided a systematic review of the fuzzy linguistic modeling approaches developed over the last decade. The reviewed methods are classified into six categories based on different approaches. In addition, recently, Bustince et al. [5] have focused on the history, definition, and basic properties of fuzzy set types and relationships between the different types of fuzzy sets.

In last couple of years, many researchers also proposed different functions for intuitionistic fuzzy sets (IFSs) and applied them in various real-time applications. In 1983, IFS was introduced by Atanassov [1] as generalization of fuzzy sets. Basically intuitionistic fuzzy sets based models may be adequate in situations when we face human testimonies, public opinions, etc. IFSs can be viewed as a generalization of fuzzy sets that may better model imperfect information which is present in any conscious decision-making (Atanassov [2]). Intuitionistic fuzzy sets take into account both the degrees of membership and non-membership on the real unit interval [0, 1] subject to the condition that their sum belongs to the same interval. In recent years, several researchers extended IFS based on various decision-making techniques.

For the first time, De Luca and Termini [11] integrated the entropy concept (Shannon [15]) with fuzzy set theory (Zadeh [42]). The main purpose of entropy measures is to explain uncertainty degree. In recent years, numerous studies have integrated the entropy with various fuzzy sets types, such as; Burillo and Bustince [4], Coban [9], Joshi and Kumar [21], Yari et al. [38, 39], Szmidt and Kacprzyk [26], Farnoosh et al. [14], Ye [40], Hung and Yang [18], Rahimi and Kumar [28], Rahimi et al. [29], Wei et al. [34], Zeng and Li [46], Szmidt and Kacprzyk [27], Ye [41], and Zhang et al. [47]. Burillo and Bustince [4] have defined the interval-valued fuzzy sets and IFSs, and introduced the distance measure between IFSs using the entropy measures. Joshi and Kumar [21] introduced the novel parametric (R, S)-norm intuitionistic fuzzy entropy for solving problem of multiple-attribute decision-making (MADM). Szmidt and Kacprzyk [26] have proposed the new non-probabilistic-type entropy measure for IFSs by considering IFSs and a ratio of distance between them. Ye [41] has introduced the fuzzy cross entropy based on interval-valued intuitionistic fuzzy sets (IVIFSs) using the intuitionistic fuzzy (IF) cross entropy. Hung and Yang [18] have applied the probability concept for introducing the fuzzy entropy IFSs using two entropy measures for IFSs. Wei et al. [33] have introduced the entropy measure for IVIFSs by incorporating three kinds of entropy measures, and, finally, proposed the new entropy measure for IVIFSs.

Some researchers have used the entropy and IFSs in various application areas such as supplier and vendor selection (Shahrokhi et al. [24], Wen et al. [35], Gerogiannis et al. [16], Xiao and Wei [37], Wang and Lv [31], Krishankumar et al. [22], Song et al. [25], Guo et al. [17], Chai et al. [6], Bali et al. [3], Wen et al. [35], and Xiao and Wei [37]). Shahrokhi et al. [24] have proposed the integrated approach based on IFS and linear programming technique for selection of suppliers in a group decision-making environment. Wen et al. [35] have considered the IFS for selection of vendor based on some MADM approaches such as Simple Additive Weighting (SAW), Weight Product Matrix (WPM), ELimination Et Choix Traduisant la REalité – Elimination (ELECTRE), Order of Preference by Similarity to Ideal Solution (TOPSIS), and Lexicographic. Gerogiannis et al. [16] have introduced the hybrid approach for assessment of biomass suppliers by integrating IFS, multi-periodic optimization (MPO), and linear programming. Wang and Lv [31] have investigated induced intuitionistic fuzzy Einstein hybrid aggregation operator (I-IFEHA) for selection of supplier in environment of group decision-making based on fuzzy measures by introducing aggregation and Einstein operator I-IFEHA. Krishankumar et al. [22] have introduced a novel approach for supplier selection using IVIF based on statistical variance (SV) and ELECTRE methods. Wen et al. [35] have used IFS for supplier selection in environment of group decision-making. Xiao and Wei [37] have presented a method to deal with the supplier selection problem in supply chain management with interval-valued intuitionistic fuzzy information. It may, however, be noted that although, these researchers have applied and integrated entropy with IFSs in various application areas, but there are gaps in application of these techniques in supplier selection. Therefore, in this paper, we have focused on to propose the new intuitionistic fuzzy entropy measure for selection suppliers.

The paper is organized as following: “Literature review” presents the literature review of entropy, IFSs, and application of these methods in assessment of supplier selection. In “Preliminaries”, we have provided some concepts and background about IFS, score function, and an Intuitionistic Fuzzy Entropy measure. A new MCDM method is proposed in “Proposed MCDM method and its application in selecting the best supplier” which discusses our case study to show the validity of the proposed method. In “Conclusion”, we conclude and state limitations and recommendations for future studies.

## Literature review

In recent decades, several of previous studies used, integrated, and introduced the entropy and IFS in numerous application areas. Burillo and Bustince [4], defined the interval-valued fuzzy sets and IFSs and introduced the distance measure between IFSs using the entropy technique. Wen et al. [35] used the IFS for selection of vendor based on some MADM approaches such as Simple Additive Weighting (SAW), Weight Product Matrix (WPM), ELimination Et Choix Traduisant la REalité—Elimination (ELECTRE), Order of Preference by Similarity to Ideal Solution (TOPSIS), and Lexicographic. Wang et al. [32] extended some operators including triangular intuitionistic fuzzy ordered weighted averaging (TIFOWA), triangular intuitionistic fuzzy ordered weighted geometric (TIFOWG), hybrid weighted averaging (IFHWA), triangular intuitionistic fuzzy generalized ordered weighted averaging (TIFGOWA), and triangular intuitionistic fuzzy generalized hybrid weighted averaging (TIFGHWA) based on TOPSIS and multi-objective programming. Shahrokhi et al. [24] proposed the integrated approach based on IFS and linear programming technique for selection of suppliers in a group decision-making environment. Joshi and Kumar [21] introduced the novel parametric (R, S)-norm intuitionistic fuzzy entropy for solving problem of multiple-attribute decision-making (MADM). Jin et al. [20] proposed two new approaches for group decision-making to derive the normalized intuitionistic fuzzy priority weights from IFPRs based on multiplicative consistency and the order consistency. Gerogiannis et al. [16] introduced the hybrid approach for assessment of biomass suppliers by integrating IFS, multi-periodic optimization (MPO), and linear programming. Wang et al. [32] proposed the new method by integration OWA–TOPSIS and intuitionistic fuzzy settings. Chen and Chang [7] proposed novel approach for fuzzy multiattribute decision-making based on three operators named IFWGA, IFOWGA, and IFHGA. Wang and Lv [31] investigated induced intuitionistic fuzzy Einstein hybrid aggregation operator (I-IFEHA) which is investigated for selection of supplier in environment of group decision-making based on fuzzy measures by introducing aggregation and Einstein operations for proposing the I-IFEHA. Szmidt and Kacprzyk [26] proposed the new entropy measure for IFSs in the non-probabilistic-type by interpreting of IFSs and a ratio of distance between them. In 2007, Vlachos and Sergiadis [30] proposed the intuitionistic fuzzy divergence measure for the first time, and studied its application pattern recognition and medical diagnosis. Krishankumar et al. [22] introduced the novel approach for supplier selection using IVIF based on statistical variance (SV) and ELECTRE methods. Ye [41] introduced the fuzzy cross entropy based on interval-valued intuitionistic fuzzy sets (IVIFSs) using the intuitionistic fuzzy (IF) cross entropy. Furthermore, Wei and Ye [34] proposed an improved version of intuitionistic fuzzy divergence in Vlachos and Sergiadis [30] and developed a method for pattern recognition with intuitionistic fuzzy information. Wen et al. [35] used IFS for supplier selection in environment of group decision-making. Hung and Yang [18] used the probability concept for introducing the fuzzy entropy IFSs with two entropy measures for IFSs. Hung and Yang [19] defined another divergence measure called ‘J-divergence’ for measuring the difference between two IFSs and then applied it to clustering analysis and pattern recognition. Burillo and Bustince [4] introduced the concept of entropy in intuitionistic fuzzy set theory, which allows us to measure the degree of intuitionism associated with an IFS. Vlachos and Sergiadis [30] proposed another measure of intuitionistic fuzzy entropy and revealed an intuitive and mathematical connection between the notions of entropy for fuzzy set and intuitionistic fuzzy set. Wei et al. [33], introduced the entropy measure for IVIFSs by incorporating three kinds of entropy measures, and finally, proposed the new entropy measure for IVIFSs. Zhang and Jiang [48] defined a measure of intuitionistic fuzzy entropy for intuitionistic fuzzy sets by generalizing of the De et al. [10], logarithmic fuzzy entropy. Xiao and Wei [37] presented a method to deal with the supplier selection problem in supply chain management with interval-valued intuitionistic fuzzy information. Although, previous mentioned papers have investigated the important role of entropy and IFS in assessment of supplier selection, but there is gap in literature regarding to these issues, however; this study based on current literature, attempted to review these issues comprehensively.

## Preliminaries

Some basic definitions of IFS, Intuitionistic Fuzzy Entropy measure, and the score function are reviewed for the sake of completeness.

### Definition 1

(Atanassov [2]). An IFS over *X* is defined as follows:

$$\begin{aligned}\tilde{A}& =\left\{\langle x,{\mu }_{\tilde{A}}\left(x\right),{\gamma }_{\tilde{A}}\left(x\right)\rangle |x \in X\right\}\\ &{\mu }_{\tilde{A}}\left(x\right) :X\to \left[\mathrm{0,1}\right], \quad {\gamma }_{\tilde{A}}\left(x\right) :X\to \left[\mathrm{0,1}\right],\end{aligned}$$

where *µ* and *γ*, respectively, define the degree of membership and the degree of non-membership, and we have: $0\le {\mu }_{\tilde{A}}\left(x\right)+{\gamma }_{\tilde{A}}\left(x\right)\le 1$ $\mathrm{for every }x \in X$.

${\uppi }_{\tilde{A}}=1-{\mu }_{\tilde{A}}\left(x\right)-{\gamma }_{\tilde{\mathrm{A}}}\left(x\right)$ denotes a measure of non-determinancy which is called the intuitionistic fuzzy (IF) index of the element *x*. Obviously, when ${\mu }_{\tilde{A}}=0$, the set $\tilde{A}$ is a fuzzy set. If we denote the set of all the FSs on *X* by *F*(*X*), the operations of IFSs are defined for every $\tilde{A},\tilde{B}\in F(X)$ as:

$$\begin{aligned}\bar{\tilde{A}}& =\left\{\langle x,{{\gamma }_{\tilde{A}}\left(x\right),\mu }_{\tilde{A}}\left(x\right)\rangle |\mathrm{x} \in \mathrm{X}\right\}\\  \tilde{\mathrm{A}}\wedge \tilde{\mathrm{B}} & =\left\{\langle \mathrm{x},{{\upmu }_{\tilde{\mathrm{A}}}\left(\mathrm{x}\right)\wedge\upmu }_{\tilde{\mathrm{B}}}\left(\mathrm{x}\right),{{\upgamma }_{\tilde{\mathrm{A}}}\left(\mathrm{x}\right)\vee\upgamma }_{\tilde{\mathrm{B}}}\left(\mathrm{x}\right)\rangle |\mathrm{x} \in \mathrm{X}\right\} \\ \tilde{A}\vee \tilde{B} & =\left\{\langle x,{{\mu }_{\tilde{A}}\left(x\right)\vee \mu }_{\tilde{B}}\left(x\right),{{\gamma }_{\tilde{A}}\left(x\right)\wedge \gamma }_{\tilde{B}}\left(x\right)\rangle |x \in X\right\} \\ \tilde{\mathrm{A}}\otimes \tilde{\mathrm{B}} & =\left\{\langle x,{{\mu }_{\tilde{A}}\left(x\right)+\mu }_{\tilde{B}}\left(x\right)-{{\mu }_{\tilde{A}}\left(x\right)\mu }_{\tilde{B}}\left(x\right),{{\gamma }_{\tilde{A}}\left(x\right)\gamma }_{\tilde{B}}\left(x\right)\rangle |x \in X\right\} \\ \tilde{A}\otimes \tilde{B}& =\left\{\langle x,{{\mu }_{\tilde{A}}\left(x\right)\mu }_{\tilde{B}}\left(x\right),{{\gamma }_{\tilde{A}}\left(x\right)+\gamma }_{\tilde{B}}\left(x\right)-{{\gamma }_{\tilde{A}}\left(x\right)\gamma }_{\tilde{B}}\left(x\right)\rangle |x \in X\right\} \\ \alpha \tilde{A} &=\left\{\langle x,1-{\left({1-\mu }_{\tilde{A}}\left(x\right)\right)}^{\alpha },{\left({\gamma }_{\tilde{A}}\left(x\right)\right)}^{\alpha }\rangle |x \in X\right\} \\ {\tilde{A}}^{\alpha } & =\left\{\langle x,{\left({\mu }_{\tilde{A}}\left(x\right)\right)}^{\alpha },1-{\left({1-\gamma }_{\tilde{A}}\left(x\right)\right)}^{\alpha }\rangle |x \in X\right\}.\end{aligned}$$

### Definition 2

(Wu-Zhang [36]). Let $\tilde{A}=\left\{{\tilde{a}}_{1},{\tilde{a}}_{2}, \dots , {\tilde{a}}_{n}\right\}$ be an IFS and ${\tilde{a}}_{i}=\left({\mu }_{i},{\gamma }_{i}\right)$, *i* = 1,2,…,*n*, be intuitionistic fuzzy values in $\tilde{A}.$ Then, an Intuitionistic Fuzzy Entropy measure is formulated in the following way:

1 $$\varepsilon \left({\tilde{a}}_{i}\right)={\pi }_{i}-{\left(\mathrm{Ln}2\right)}^{-1}\left[{\mu }_{i}\mathrm{Ln}\left(\frac{{\mu }_{i}}{{\mu }_{i}+{\gamma }_{i}}\right)+{\gamma }_{i}\mathrm{Ln}\left(\frac{{\gamma }_{i}}{{\mu }_{i}+{\gamma }_{i}}\right)\right].$$

This measure satisfies the four axioms in Szmidt and Kacprzyk [24] for IF value entropy measure.

### Definition 3

(Chen and Tan [8]). Let ${\tilde{a}}_{i}=\left({\mu }_{i},{\gamma }_{i}\right)$, *i* = 1,2,…,*n*, be intuitionistic fuzzy values, and then, the score of ${\tilde{a}}_{i}$ is:

2 $$S\left({\tilde{a}}_{i}\right)= {\mu }_{i}- {\gamma }_{i},\quad i=\mathrm{1,2},\dots ,n.$$

## Proposed MCDM method and its application in selecting the best supplier

We consider that one of the largest companies in Iran would like to select the best supplier firm to provide the materials in production line. In this context, we propose a new method based on Intuitionistic Fuzzy Entropy to identify the best supplier. First, we have to recognize the main criteria which can influence our decision. After the criteria selection, the next step is how to choose the best supplier.

### Criteria selection

Using Dickson’s [13] 23 criteria in supplier selection and the addition of one local criterion which is pay off time (an important factor in Iran's business market), a questionnaire containing 24 questions was constructed. This questionnaire was sent to 30 firm’s managers and firm's sale managers. In each question, the importance of one criterion is evaluated. The applicant would choose among: “very low”, “low”, “medium”, “high”, and “very high”. All responses were converted to the five-point Likert scale. Then, using SPSS, we have compared the means of the criteria points at 95% confidence level. As follows, five criteria were selected as the most important ones such as; price, quality, deliver, technical capability, and pay off factors.

### The selection model

**Fig. 1:**
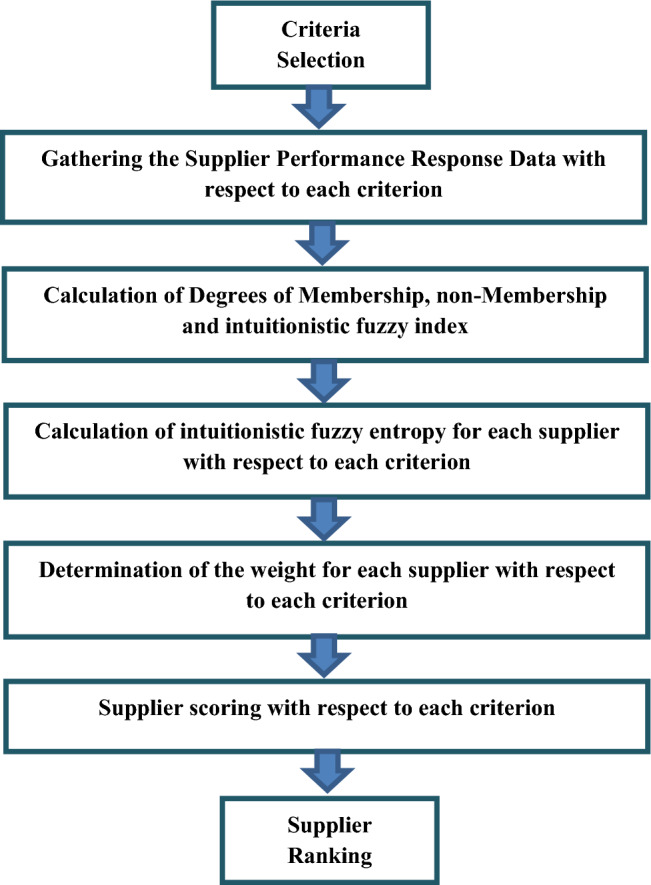
The flowchart of the criteria with respect to the suppliers

**Fig. 2:**
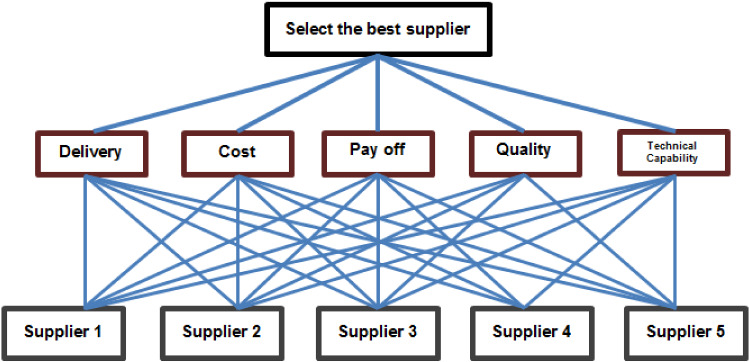
The diagram of the criteria with respect to the suppliers

In the presented selection model (shown in Fig. 1), we have tabulated the information of five suppliers in Table1 with respect to the above criteria. Note that when the values are qualitative, we convert them on the quantitative scale by the five-point Likert scale as it is shown in Table 2. That is; in the qualitative questions, the criteria with respect to each supplier are given a number among 1, 3, 5, 7 and 9 (Fig. 2).

**Table 1:** Supplier performance response data

Performance	${\mathrm{C}}_{1}$	${\mathrm{C}}_{2}$	${\mathrm{C}}_{3}$	${\mathrm{C}}_{4}$	${\mathrm{C}}_{5}$
Supplier 1	1.2	M	VH	L	1
Supplier 2	1.5	VH	H	H	3
Supplier 3	1.3	M	L	M	6
Supplier 4	1.7	H	VH	H	2
Supplier 5	1.3	H	M	H	3

**Table 2:** Supplier performance response data on five-point Likert scale

Performance	${\mathrm{C}}_{1}$	${\mathrm{C}}_{2}$	${\mathrm{C}}_{4}$	${\mathrm{C}}_{9}$	${\mathrm{C}}_{24}$
Supplier 1	1.2	5	9	3	1
Supplier 2	1.5	9	7	7	3
Supplier 3	1.3	5	3	5	6
Supplier 4	1.7	7	9	7	2
Supplier 5	1.3	7	5	7	3

To convert the values into the Intuitionistic Fuzzy Values, we have extended the method introduced in Deng-Chan [12] as follows:

To get the degrees of membership, non-membership, and intuitionistic fuzzy index, we have calculated the distance of each value with the lowest value as the value of membership, the distance of each value with the highest value as the value of non-membership, and the distance of each value with the average of others as the value of intuitionistic fuzzy index. Then, these calculated values are, respectively, divided by their total sum. If the criterion is a kind of cost, the degrees of membership and non-membership are replaced with each other. For example, the intuitionistic fuzzy degrees for the two first values of C1 are calculated here:

$$\begin{aligned} & \left\{\begin{array}{l}\frac{|1.2-1.2|}{\left|1.2-1.2\right|+\left|1.2-1.7\right|+\left|1.2-1.4\right|}=0 \quad {\mu }_{11}=0 \\ \frac{|1.2-1.7|}{\left|1.2-1.2\right|+\left|1.2-1.7\right|+\left|1.2-1.4\right|}=0.714\quad {\gamma }_{11}=0.714\\ \frac{|1.2-1.4|}{\left|1.2-1.2\right|+\left|1.2-1.7\right|+\left|1.2-1.4\right|}=0.286\quad {\pi }_{11}=0.286\end{array}\right.\\ & \left\{\begin{array}{l}\frac{|1.5-1.2|}{\left|1.5-1.2\right|+\left|1.5-1.7\right|+\left|1.5-1.4\right|}=0.50\quad {\mu }_{21}=0.333 \\ \frac{|1.5-1.7|}{\left|1.5-1.2\right|+\left|1.5-1.7\right|+\left|1.5-1.4\right|}=0.333 \quad {\gamma }_{21}=0.50 \\ \frac{\left|1.5-1.4\right|}{\left|1.5-1.2\right|+\left|1.5-1.7\right|+\left|1.5-1.4\right|}=0.167\quad {\pi }_{21}=0.167.\\ \end{array}\right. \end{aligned}$$

From the first column of Table 2, we now that Supplier 1 has the worst performance in delivery, where Supplier 4 has the best. With this proposed method, in Table 3, we see that Suppliers 4 and 1 have the highest and the lowest degrees of membership, respectively, where the Suppliers 3 and 5 also the same degrees, since they are doing the same in this criterion.

**Table 3:** The intuitionistic fuzzy values

Performance	${\mathrm{C}}_{1}$	${\mathrm{C}}_{2}$	${\mathrm{C}}_{4}$	${\mathrm{C}}_{9}$	${\mathrm{C}}_{24}$
Supplier 1	(0,0.714,0.286)	(0,0.714,0.286)	(0.714,0,0.286)	(0,0.588,0.412)	(0,0.714,0.286)
Supplier 2	(0.5,0.333,0.167)	(0.625,0,0.375)	(0.625,0.312,0.063)	(0.769,0,0.231)	(0.4,0.6,0)
Supplier 3	(0.167,0.666,0.167)	(0,0.714,0.286)	(0,0.625,0.376)	(0.417,0.417,0.166)	(0.625,0,0.375)
Supplier 4	(0.625,0,0.375)	(0.455,0.455,0.09)	(0.714,0,0.286)	(0.769,0,0.231)	(0.167,0.666,0.167)
Supplier 5	(0.167,0.666,0.167)	(0.455,0.455,0.09)	(0.263,0.526,0.211)	(0.769,0,0.231)	(0.4,0,0.6)

For intuitionistic fuzzy index in Table 3, we see that Supplier 4 has the highest value because of having the farthest distance from the average. That is, there is a better confidence for the suppliers having the value close to the average and it results to a lower intuitionistic fuzzy index.

Using Eq. (1) from Definition 2, the Intuitionistic fuzzy entropy measures are easily calculated and presented in Table 4.

**Table 4:** The intuitionistic fuzzy entropy measures

Performance	${\mathrm{C}}_{1}$	${\mathrm{C}}_{2}$	${\mathrm{C}}_{4}$	${\mathrm{C}}_{9}$	${\mathrm{C}}_{24}$	Sum	Normalized
Supplier 1	0.2857	0.2857	0.2857	0.4118	0.2857	0.9832	3.9223
Supplier 2	0.9758	0.3750	0.9234	0.2308	0.9710	3.4759	1.4296
Supplier 3	0.7683	0.2857	0.3750	1.0000	0.3750	2.5183	2.3872
Supplier 4	0.3750	1.0000	0.2857	0.2308	0.7683	2.6598	2.2457
Supplier 5	0.7683	1.0000	0.9355	0.2308	0.9710	3.9055	1.0000
Sum	3.1731	2.3750	2.5196	2.1042	3.3709		
Normalized	1.1978	1.9959	1.8513	2.2667	1.0000		

**Normalization:** The entropy measures uncertainty and it indicates that more is its value, more is uncertainty. Then, calculating the sum of each row in Table 4, the distance of each summed value of each row with the largest summed value is added to 1 and shown as normalized value (similarly for the column). The reason of adding 1 is because of the opposing behavior of the number less and more than one. For example, in the vertical group, since the biggest value is 3.9055, the first normalized value becomes:

$$ \left( {|0.{9832} - {3}.{9}0{55}|} \right) + {1} = { 3}.{9223}. $$

Now, multiplying the normalized values of each row by the normalized values of each column represents the coefficient of each criterion with respect to each supplier. For example, the coefficient of C1 with respect supplier 1 is 3.9223 × 1.1978 = 4.6981 (the difference between 4.6981 and 4.6983 is because of rounding two numbers 3.9223 and 1.1978 which are not rounded in calculations). All the coefficients are shown in Table 5. The sum of the coefficients corresponding to each criterion shows the total coefficient of each criterion. At the end, dividing each coefficient by sum of the coefficients determines its weight. Table 5 presents the sum of the coefficients of each criterion and the total weight of each criterion.

**Table 5:** Total weights of each criterion

Performance	${\mathrm{C}}_{1}$	${\mathrm{C}}_{2}$	${\mathrm{C}}_{4}$	${\mathrm{C}}_{9}$	${\mathrm{C}}_{24}$
Supplier 1	4.6983	7.8285	7.2614	8.8907	3.9224
Supplier 2	1.7124	2.8532	2.6465	3.2404	1.4296
Supplier 3	2.8595	4.7647	4.4195	5.4111	2.3873
Supplier 4	2.6900	4.4822	4.1575	5.0904	2.2458
Supplier 5	1.1978	1.9959	1.8512	2.2666	1.0000
Total	13.1579	21.9245	2.5196	24.8992	10.9850
Weight	0.1441	0.2401	1.8513	0.2727	0.1203

Finally, multiplying the weight of each criterion by the score of each intuitionistic fuzzy value which is calculated in Eq. 2 shows the importance of each criterion with respect to each supplier. For example, the importance of criterion 1 with respect to supplier 1, since its score is 0.714, is equal to 0.714 × 0.1441 = 0.1029. In Table 6, the sum of degree of importance of each supplier shows their rankings.

**Table 6:** Total rank of the criteria

Performance	${\mathrm{C}}_{1}$	${\mathrm{C}}_{2}$	${\mathrm{C}}_{4}$	${\mathrm{C}}_{9}$	${\mathrm{C}}_{24}$	Total	Ranking order
Supplier 1	0.1029	− 0.1714	0.1590	− 0.1603	− 0.0859	− 0.0859	4
Supplier 2	− 0.0241	0.1501	0.1392	0.2097	− 0.0241	0.4508	1
Supplier 3	0.0721	− 0.1714	− 0.1392	0.0000	0.0752	− 0.1634	5
Supplier 4	− 0.0901	0.0000	0.1590	0.2097	− 0.0602	0.2185	2
Supplier 5	0.0721	0.0000	− 0.0586	0.2097	0.0241	0.1991	3

From Table 6, it is noted that the values of total rank of criterion for the suppliers are − 0.0859, 0.4508, − 0.1634, 0.2185, and 0.1991, respectively. Thus, the selection preferences of suppliers may be stated as:

$$ {\text{Supplier 2}} > > {\text{Supplier 4 }}> > {\text{Supplier 5}} > >  {\text{Supplier 1}} > > {\text{Supplier 3}}, $$

indicating that Supplier 2 is the best.

## Conclusion

In this investigation, we have introduced a new entropy-based model which extends the notion of intuitionistic fuzzy sets. To show the applicability of the proposed method, we have considered the problem of selecting the best supplier firm to provide the materials in production line of a large company in Iran. For economic considerations, every company wants to use a method of decision-making to select the best supplier. Obviously, criterion based only on expertise is infeasible some time. By the use of intuitionistic fuzzy entropy, we have attained a new method to provide a standard measurement to select the best supplier. In literature, earlier researchers have demonstrated that expertise had a strong effect in the selection of best supplier especially in determining the range of the weight. However, in our proposed, novelty lies in the fact that a standard method is applied for determination of the weight and wherein the expertise effect on the decision-making has been reduced, thus, making the proposed method more applicable.

In continuation for the future work, we are going to construct the matrix of the optimal weights based on the intuitionistic fuzzy entropy values for decision-makers with respect to the attributes of the alternatives. Then, based on this matrix of weights, and some operators such as weighted averaging operator and the score function, the rank of the suppliers will be denoted by the scores which they gain. As a hint for other authors, the method provided in this paper can also be used in portfolio optimization when the calculated weights can represent the share of each stock.
